# Supplementary material for: Use of a filamentous green alga (Chaetomorpha sp.) and microsnail (Stenothyra sp.) as feed at an early stage of intensive aquaculture promotes growth performance, artificial feed efficiency, and profitability of giant tiger prawn (Penaeus monodon)
Source: PLoS One. 2020 Dec 31;15(12):e0244607. doi: 10.1371/journal.pone.0244607 (PMC7774951; doi:10.1371/journal.pone.0244607)
Supplement: Attachment — Submitted filename: Response to Reviewers.docx [file pone.0244607.s001.docx]

Mahmoud A.O. Dawood, PhD

Academic Editor

*PLOS ONE*

9 November 2020

Submission no.: PONE-D-20-21866

Title: Use of a filamentous green alga (*Chaetomorpha* sp.) and microsnail (*Stenothyra* sp.) as feed at an early stage of intensive aquaculture promotes growth performance, artificial feed efficiency, and profitability of giant tiger prawn (*Penaeus monodon*)

**Dear Dr. Dawood,**

**Thank you very much for your valuable insights. We are delighted that you think our work will spark discussion in our field. We are grateful for the time and energy you have expended on our behalf.**

**Below, you will find our responses (in bold text) to each of your and the reviewers’ questions and comments. Also, all of the revisions made to our original manuscript appear in red texts (deletions with strick-out lines, additions with only red color texts) in the file titled “revised manuscript with tracked changes”.**

**Response to Reviewer #1**:

**The authors are very appreciate for helpful comments.**

Abstract: It is hard to get to know the main content of this manuscript form the abstract. Author should provide more effective information in concise sentences.

**Following the comments from Reviewers #1 and #4, we have reduced the amount of background information in the abstract of the revised manuscript and inserted more details on the methodology and results.**

**We hope that the abstract has been improved to your satisfaction, in terms of conciseness and readability.**

Line 27-31: Confused. Please rewrite it.

**We have emphasized the point “benthic organisms were propagated together with *P. monodon* post-larvae” on L34–36 of the revised manuscript tracked changes.**

Line 32-37: Rewrite. More results should be exhibited in abstract.

**We have added the values associated with our key results, such as the individual shrimp weight, survival rate, shrimp production, artificial feed conversion ratio, shrimp sales, and balance between shrimp sales and costs. These details can be found on L47–51 of the revised manuscript tracked changes.**

Introduction:

Line 69: References are needed here.

**We have inserted several references on L89 of the revised manuscript tracked changes.**

Line 75-82: Redundant content. Why you choose Chaetomorpha sp. and Stenothyra sp.?

**We used these benthic organisms because they were “considered promising candidates as supplementary feeds for tropical Penaeidae”, as mentioned on L78–79 of the original manuscript. Also, we think that it is necessary to include some brief information on these benthic organisms to help readers better understand them. Therefore, we would prefer to keep this paragraph. We hope this adequately answers your question. If not, please let us know.**

Line 84-85: Why culture both Chaetomorpha sp. and Stenothyra sp. as supplementary live feeds? Why use them as feed at an early stage of intensive aquaculture?

**We used these benthic organisms as supplementary feed because both benthic species promote shrimp growth, as mentioned in L79–80 of the original manuscript.**

**These benthos were used as feed only at an early stage of culture to maintain a low level of additional operating efforts, as stated in L87–89 of the original manuscript.**

**To improve and make this paragraph easier to understand, we have added the following sentence highlighting our main objective in this study (“The aim of this study was to develop …. findings advance ….”) at the beginning of the paragraph (L105–107 of the revised manuscript tracked changes).**

**We hope our revisions are satisfactory.**

Materials and methods:

Line 120: Why choose these amounts?

**This is also related to the question raised above. The amounts of the benthic organisms were limited because we wanted them to be completely consumed within 2 months, to keep operating efforts low, as described on L87–89 of the original manuscript.**

Line 121-123: How to remove other benthic organisms?

**Other organisms rarely occurred because we thoroughly washed all benthic organisms with freshwater during preparation (please refer to L109 of the original manuscript), but we tried to regularly monitor the underwater situation and removed other organisms by hand, siphon, or filtration (depending on the situation).**

**We have added this information on L152–153 of the revised manuscript tracked changes.**

Line 123-124: How about the daily management?

**We assume that “daily management” here refers to the daily management of the feed amount. If our understanding is correct, we managed the amount of feed being offered each day using the feeding tray method. As mentioned in L125–128 of the original manuscript, “The amount of artificial feed provided was determined via the feeding tray method, in which the feed was adjusted to minimize the amount remaining after each feed. This was accomplished by recording the amount of feed remaining on the feeding tray in each pond and using it as an indicator of the appetite of the shrimp”.**

Line 134: Only 20 shrimps? Compared with the initial number of shrimps, the measured quantity is quite small, whether it leads to a big error?

**We appreciate your helpful comment on the statistical analysis used in our study. We would like to explain our rationale regarding this matter.**

**For this series of growth measurements, 20 post-larvae were randomly sampled from each pond (3 control ponds and 3 experimental ponds) and their individual wet weights were measured. Approximately 2,700 individuals were cultivated in each pond. This means a total 60 individuals (randomly sampled) out of 8,100 individuals (from the 3 ponds) were individually weighed from each treatment every month.**

**Using a software program called "G*Power", we calculated the statistical power of the above design to be more than 90%. For example, in the first month:**

**Mean value in treatment: 1.435 g (SD: 0.895 g)**

**Mean value in control: 0.795 g (SD: 0.34 g)**

**Power: 0.99 (99%)**

**In months 2 and 3, the observed power values were also both 0.99. These values greatly exceed 0.8, which is the power value that is generally considered acceptable.**

**Given the abovementioned random sampling design and calculated power values, we consider our sample size of 20 individuals from each pond to be sufficient for making comparisons between the control and treatment.**

Discussion：

Line 280-283: Confused. Please explain these sentences.

**As Reviewer #2 also commented, “The discussion of IMTA on page 18 is superfluous; as the authors state, this study is not an example of integrated aquaculture, it is the addition of live feeds.” Therefore, we have deleted this section (L397–410 of the revised manuscript tracked changes).**

Table 1: How do you calculate the apparent benthos consumption?

**We calculated “apparent benthos consumption” as the difference in benthos biomass between any sampling time and one previous sampling time. We have added this formula at the end of the subsection titled “Measurement of benthos biomass change and apparent benthos consumption” (L208–212 revised manuscriot tracked changes).**

Table 3: There are no Chaetomorpha sp. Stenothyra sp. provided after 8 weeks. Why the higher feed consumption was found in experimental treatment?

**Because the shrimp in the experimental treatment ponds grew faster (presumably due to benthos consumption) by 8 weeks, the larger shrimp in the treatment ponds likely consumed more artificial shrimp feed than the shrimp in the control ponds did.**

Table 4: 0 may not possible. Please explain.

**Thank you so much for pointing out this error. We have changed the values using the appropriate decimal places in Table 4 of the revised manuscript tracked changes.**

Table 5: What is the amount of benthos? What is the amount of benthos? Line 120: 6.81 kg and 1.96 kg. Survival rate>100%???

**Our experimental ponds had a volume of 97.2 m^3^ (9 m wide × 9 m long × 1.2 m deep). The amounts of *Chaetomorpha* and *Stenothyra* were 6.81 kg (6,810 g) and 1.96 kg (1,960 g), respectively, in each treatment pond. Therefore, the unit amount (per m^3^) of *Chaetomorpha* was 6,810 g / 97.2 m^3^ = 70.06 (~70 g m^-3^), and the unit amount (per m^3^) of *Stenothyra* was 1,960 g / 97.2 m^3^ = 20.16 (~20 g m^-3^)**

**As for the survival rates, shown here are the ratios of the survival rate in the treatment compared with the control (100%). These are presented as the “ratio of best value in treatment to control” in column 9. This is why the survival rate value was higher than 100%. The survival rates of Penaeidae from six past reports are also presented this way, as the ratio of treatment to control survival.**

**Response to Reviewer #2**:

**Thank you very much for the many constructive comments.**

Apparently, the experimental diet stimulated growth; the performance of P. monodon appears to have improved in the 3 groups given supplemental feeds as PLs as compared with controls. This comparison is limited statistically by the small number of groups (N = 3) and apparent variation in food consumption and survival. For these reasons I suggest that this be correctly described as as a pilot or preliminary observation and not presented as a definitive analysis. When an experimental treatment appears to hold the promise of increasing commercial production and improved profitability for a valuable crop, is is reasonable to test it on a scale that is adequate for the use of rigorous statistical analysis.

**Thank you very much for your constructive comment regarding the sample sizes (*n* = 3) used to study shrimp production, feed efficiency, and profitability. We would like to express our views on this matter.**

**One of our main purposes in this study was to evaluate profitability (shrimp sales, artificial feed cost, miscellaneous costs, and the balance between shrimp sales and costs). These data can be obtained only from large-scale experiments, because small-scale experiments (e.g., experiments using small tanks in the laboratory) cannot generate large numbers of shrimp, nor can they accurately evaluate shrimp profitability. The final data collected at harvest and mid-buyer are summarized in Table 1. The mean values and standard deviations from the control ponds (*n* = 3) and experimental ponds (*n* = 3) are shown for each factor measured, and t-tests were applied to compare the control and experimental treatment data.**

**According to the profitability data for “shrimp sales” in Table 1,**

**Mean value for treatment: 215.97 USD (SD: 4.37 USD)**

**Mean value for control: 155.73 USD (SD: 10.27 USD)**

**Power: 0.999 (99.9%), calculated with a software program called "G*Power"**

**The power value of shrimp sales greatly exceeds 0.8, which is the generally required power value. In addition to this, other factors related to profitability (shrimp feed cost: 0.81, and balance of sales and costs: 0.99) also exceed 0.8. This indicates that the profitability data are sufficient to evaluate the statistical difference between the control and treatment under the *n* = 3 condition.**

**For an alternative viewpoint, according to the “final mean individual weight (g WW)” in Table 1,**

**Mean value for treatment: 18.2 g (SD: 1.07 g)**

**Mean value for control: 16.0 g (SD: 0.61 g)**

**Power: 0.627 (62.7%), calculated with a software program called "G*Power"**

**Although the calculated power is slightly lower than the generally required power value (0.8), we think that this value is still within an acceptable range, and a significant difference was in fact detected. Therefore, for the "final mean individual weight", we think that the significant difference (p = 0.037) between the control and experimental treatment is valid, even under the condition of *n* = 3 and the lower calculated power value.**

**The power values of the other factors in Table 1 vary, but all of them exceed 0.6 (meaning they are within the acceptable range) except for “initial mean individual weight” (starting shrimp weight). The power values of the various factors (shown in Table 1) used to compare the control and experimental treatments are as follows:**

**Specific growth rate: 0.65**

**Total number of shrimp produced: 0.60**

**Survival rate: 0.60**

**Total shrimp production: 0.99**

**Apparent artificial feed intake: 0.80**

**Calculated artificial feed protein intake: 0.81**

**Artificial feed conversion ratio: 0.61**

**Artificial shrimp feed cost: 0.81**

**Shrimp sales: 0.99**

**Balance between shrimp sales and costs: 0.99**

**As Reviewer #2 mentioned, the sample size (*n* = 3) used in this study seems small, but considering the abovementioned power values for the factors in Table 1, our sample size was sufficient to make comparisons between the control and experimental treatments.**

Was any cost or value ascribed to the green algae and snails that were used as supplemental feeds in this study? It is not clear to me if these organisms would be available to scale up for culture on a commercial scale. The experimental group ate more of the prepared commercial feed, so at least one mechanism of action was an increased appetite in response to the early supplemental feeds.

**We appreciate these important questions.**

**Initially, we did not consider the costs of benthos collection. The reasons are that *Chaetomorpha* and *Stenothyra* “grow abundantly in stagnant waters and/or channels in mangrove areas along tropical coasts” (please see L81–82 of the original manuscript). These benthic organisms are familiar species that can be easily collected around shrimp culture areas, which are usually located around mangroves. This study mainly focused on small-scale shrimp farmers, who operate family-run shrimp culture operations. Accordingly, the benthos collection procedures in this study were designed to be very minimal. More specifically, “the benthos amounts collected …. were minimized to the extent that all were consumed within ~1–2 months, to maintain a low level of additional operating efforts.” (L87–89 of the original manuscript). The benthos collection period was also limited to “3–4 h” (L107–108 of the original manuscript). Therefore, we assumed that the costs associated with benthos collection would be very little, if any.**

**However, as Reviewer #2 correctly mentioned, there may be owners who run shrimp culture operations alone. For such cases, we have added “miscellaneous costs”, which include personnel (to help with benthos collection) and fuel costs, to Table 1. These costs were calculated using the actual costs from our experiment (three helpers for 4 hours and fuel).**

**Regarding miscellaneous costs, we added “and miscellaneous costs for benthos collection” on L514 of the revised manuscript tracked changes. We also changed the balance values in Table 1, the abstract (L50–51), results (L302–303, L 365–371), discussion (L514), and conclusion (L546) of the revised manuscript tracked changes.**

**As for whether “these organisms would be available to scale up for culture on a commercial scale”, we have also considered this and agree that it is important. To apply this system to real earthen ponds in the future, simple techniques for self-propagating benthos around or inside earthen culture ponds must be developed so that less benthos collections are required. In fact, this is the next experimental step for us. We have added two sentences addressing this on L527–532 in the revised manuscript tracked changes.**

The discussion of IMTA on page 18 is superfluous; as the authors state, this study is not an example of integrated aquaculture, it is the addition of live feeds.

**We have deleted this entire subsection from the discussion (L397–410 of the revised manuscript). In relation to this, the reference numbers have been changed on L463, L503, L517, and L518–519 of the revised manuscript tracked changes.**

The abstract refers to the culture of “an early stage” of P. monodon; it would be preferable to identify them specifically as P. monodon post larvae.

**We have modified the abstract based on the comments from reviewers #1 and #2.**

Line 217 Penaeus monodon should be italicized for correct scientific name format.

**We have changed “Penaeus monodon (normal text)” to “*Penaeus monodon* (italic text)” (L268–269 of the revised manuscript tracked changes). Thank you so much for pointing this out.**

The different survival rates with such a low number of samples (3 control and 3 experimental ponds) is troubling – as the authors mentioned more than once, this experimental design does not allow for strong and definitive statistics. With such a small number of samples, random variation or tank effects cannot be ruled out.

**Because the statistical power value for survival rate was 0.6 (within an acceptable range) as mentioned above, we believe that the sample size was sufficient to make comparisons between the control and experimental treatments.**

**Response to Reviewer #3**:

**We appreciate your valuable comments on our manuscript.**

Table 1. check your value.

If 16.0 ± 6.1 is correct, it might be strongly possible that the statistics also are subject to change. 6.1 is high so I didn't see a statistical difference between 16.0 and 18.2.

**The SD of 6.1 shown on row 4 (final mean individual shrimp weight) of the “control” column was incorrect owing to an oversight on our part. The correct value is 16.0 ± 0.61, and this was calculated from the control data (control 1: 16.2 g, control 2: 15.4 g, control 3: 16.6 g), which give a mean value of 16.0 g and SD of 0.61. We have corrected this SD value with the appropriate decimal places. Thank you so much for catching this critical error.**

Figures.

The figures are low resolution, which makes their observation difficult. I suggest increasing the resolution.

**Following the suggestion in the *PLOS ONE* guidelines, we uploaded all of our figure files using the Preflight Analysis and Conversion Engine (PACE) digital diagnostic tool (https://pacev2.apexcovantage.com/), which helps ensure that figures meet *PLOS ONE* requirements. All of our figures met the journal’s requirements, according to the PACE site. Thus, it is possible that the low resolution of figures arose from the compression of files for review purposes.**

**Response to Reviewer #4**:

The authors are encouraged to address the following issues:

**We greatly appreciate your comments.**

1- The article language needs edition.

**We will carefully edit all of the English throughout our manuscript.**

2- Abstract needs restructuring. It contains much background data and less methodological data and results. Revise it.

**We have reduced the background description and added more details on our methodology and results.**

3- It is not clear if the added live food reproduced in the culture tank during 15 weeks of experiment. Clarify it.

**Actually, we performed frequent underwater observations during the experimental period, but we did not find any small microsnails. This likely indicates that *Stenothyra* sp., did not reproduce during the experimental period. For the green alga, *Chaetomorpha* sp., we cannot definitely state whether *Chaetomorpha* sp. reproduced in the culture ponds during the experimental period. However, the experimental ponds were dark, so we assumed that this green alga would be unable to grow, mature, and reproduce. Even if reproduction did occur, only small amounts of algae would have been produced, and these amounts could be omitted.**

4- In table 1, "total shrimp production": Unit is confusing. kg shrimp per tank? per squared meter?... Moreover, add feed intake as percentage of body weight.

**Almost all of the values in Table 1 are shown as the means and standard deviation for each of the two treatments (*n* = 3). These mean values are thus “per pond”, except for “productivity (kg WW m^-2^)”. To make this clearer, we have inserted “*n* = 3” in the column headings and changed the wording of the table legend from “Values are the means ± SD of three replicates” to “Values shown are the means ± SD for three replicate ponds”. Additionally, we deleted the row labeled “productivity (kg WW m^-2^)” and “…and productivity were…” from L 282 of revised manuscript with track changes, because this factor is essentially the same as “total shrimp production (kg WW)”.**

**Regarding the suggestion of adding “feed intake as percentage of body weight,” this is already expressed through the “artificial feed efficiency (%),” which is a reciprocal of the artificial feed conversion ratio (aFCR). Because the values for aFCR already appear in the table, we would prefer not to add the suggested metric, to avoid possible confusion and overlap of data.**

**Thank you so much to the editor and all of the reviewers for their important comments.**

**In addition to the reviewers’ comments, we have found and corrected some errors in the manuscript, as follows.**

**Table 1**

**3^rd^ row: (error) 2.0 ± 0.0 ^a^ → (correct) 2.0 ± 1.0 ^a^**

**5^th^ row: (error) 7.33 ± 0.12 ^a^ → (correct) 8.15 ± 0.05 ^a^**

**(error) 7.59 ± 0.08 ^b^ → (correct) 8.41 ± 0.01 ^b^**

**9^th^ row: deleted due to overlap with “total shrimp production” (8^th^ row)**

**11–16^th^ row: added “N/A” to blank cells (control column)**

**21–24^th^ row: added more decimal places to show exact values**

**Table 3**

**5–7^th^ row: added “N/A” to blank cells**

**L364–371 of revised manuscript tracked changes: checked and corrected USD values with two decimal places**

**L563 of revised manuscript tracked changes: (error) Ohono → (correct) Ohno**

**Thank you again for your insightful comments and for providing us with the chance to revise our manuscript. We look forward to hearing from you regarding our submission. Please do not hesitate to contact me with any further questions and comments that you may have.**

**Kind regards,**

**Isao Tsutsui**

**Fisheries Division, Japan International Research Center for Agricultural Sciences (JIRCAS)**

1-1 **Ohwashi, Tsukuba, Ibaraki 305-8686, Japan**

**Phone: +81-(0)29- 838-6608**

**Fax: +81-(0)29- 838-6655**

**E-mail: cong@affrc.go.jp**
